# Age, Age‐Related Comorbidities and Survival in Palbociclib, Ribociclib and Abemaciclib Users With Advanced Breast Cancer: A Nation‐Wide Retrospective Cohort Study

**DOI:** 10.1002/pds.70416

**Published:** 2026-06-16

**Authors:** Blair Rajamaki, Olli Tenhunen, Liisa Pylkkänen, Thuan Vo, Anne Paakinaho, Miia Turpeinen, Sirpa Hartikainen, Anna‐Maija Tolppanen

**Affiliations:** ^1^ School of Pharmacy University of Eastern Finland Kuopio Finland; ^2^ Finnish Medicines Agency Helsinki Finland; ^3^ Medical Research Center Oulu University of Oulu and Oulu University Hospital Oulu Finland; ^4^ Kuopio Research Centre of Geriatric Care University of Eastern Finland Kuopio Finland

**Keywords:** anti‐cancer drugs, breast cancer, geriatric medicine, pharmacoepidemiology, prescribing

## Abstract

**Purpose:**

Prevalence of breast cancer (BC) increases with age, but the external validity of data obtained from pivotal trials of medicinal products remains a concern due to the underrepresentation of frail and older adults. Cyclin‐dependent kinase inhibitors (CDKi), palbociclib, abemaciclib, and ribociclib are considered an essential part of the standard‐of‐care in the management of advanced/metastatic breast cancer. We investigated age, comorbidities, and survival in a nationwide real‐world cohort of CDKi users.

**Methods:**

Data from the Finnish Cancer Registry, reimbursed dispensed prescriptions, Electronic Prescription Database, Care Register for Health Care (CRHC), and Causes of Death Register were combined and analysed.

**Results:**

Altogether 1921 women with BC initiated CDKi treatment in 2018–2022. The median age at initiation was 66.9 years, with 43.2% of the study cohort being < 65 years of age, 36.1% 65–74 years, 19.0% 75–84 years, and 1.7% 85 years or older. The prevalence of age or frailty‐related comorbidities in individuals ≥ 65 years was low, highest for cardiovascular diseases, 32.3%, and diabetes, 16.9%. The median survival was 25.3 months, with longer median survival times in younger age groups (27.7 months in < 65 year‐olds, 25.1 months in aged 65–74, 21.4 months in aged 75–84 years, and 15.4 months in 85 years or older).

**Conclusions:**

Real‐world CDKi users are older than women included in the pivotal trials, and the survival differences between age groups imply challenges in the generalisability of data from pivotal trials. The real‐world CDKi user population is characterised by a low prevalence of major comorbidities.

## Summary

- This study addresses knowledge gaps in the use of CDKis, the current standard‐of‐care in the pharmacotherapy of advanced breast cancer based on comprehensive registry data.
- CDKis are used for advanced or metastatic breast cancer in older women than those who were included in the pivotal trials of these drugs.
- Oldest age groups of CDKi users show an inferior overall survival compared to younger groups in the real‐world setting.
- Older breast cancer patients using CDKis are characterised by few age‐ or frailty‐related comorbidities.
- The findings may imply challenges in the generalisability of data from pivotal breast cancer trials in older women, while alleviating the concerns on external validity in terms of frailty.

## Plain Language Summary

This study reports the real‐world patient characteristics, age‐specific survival and concomitant illnesses of users of cyclin‐dependent‐kinase inhibitor drugs (CDKi), the currently recommended standard treatment of incurable breast cancer. In routine clinical practice, users of CDKis are older than those included in the pivotal clinical trials where these drugs have initially been studied. The oldest age groups have an inferior survival compared to younger patients. CDKi users have few other major age‐related diseases or conditions. The results of this study may imply challenges in the generalisability of results from previous breast cancer studies in older women.

## 1 Introduction

Incidence and prevalence of breast cancer (BC), the most common malignancy in women, increase with age, with more than one‐third of the cases diagnosed in patients aged over 70 years [1, 2, 3, 4]. While advancing age is often considered to be associated with a more favourable tumour biology, and BC specific survival in older women has been suggested to be similar to survival in the general population irrespective of disease status, real‐world data have suggested an inferior overall survival (OS) of older adults with BC [5, 6].

Hormone‐receptor (HR) positive BC represents the largest subtype of BC, accounting for 60%–65% of all cases, and available data suggest increased estrogen receptor (ER) positivity and decreased human epidermal growth factor receptor (HER)‐2 expression with increasing age [5, 7]. For decades, the treatment of HR positive advanced BC has been based on the inhibition of the ER pathway, until the introduction of cyclin‐dependent kinase inhibitors (CDKis), palbociclib, abemaciclib, and ribociclib, transformed its clinical management [7, 8, 9]. CDKis, in combination with aromatase inhibitors or fulvestrant, are considered a current standard of care in the management of advanced or metastatic BC in post‐menopausal patients irrespective of age [10, 11]. CDKis have been initially demonstrated to improve progression‐free survival (PFS) in advanced BC with subsequent analyses to show an improvement in the OS, and compared with conventional chemotherapy agents, they are generally considered to have a manageable safety profile [10, 11, 12].

The evidence and guidelines to treat older cancer patients primarily rely on subgroup analyses, and extrapolation of efficacy and safety data from younger individuals. At the same time, underrepresentation of older adults in anti‐cancer drug development is well‐recognised [1, 13]. Anti‐cancer medicinal products are most commonly authorised and enter the market based on a single pivotal clinical trial, a regulatory pathway which was also followed at the initial marketing authorisations of CDKis [6, 7, 8]. The underlying reasons for the global underrepresentation of older adults in anti‐cancer drug development are considered multifactorial, yet low accrual and exclusion of these patients from trials have been suggested as main causes [1].

Older adults, including patients with BC, are a heterogenous patient group that also has an increased prevalence of comorbidities that may impact life expectancy, cancer prognosis, and treatment tolerance [14, 15]. More importantly, these comorbidities may have a direct impact on the types of treatments that can and should be offered [1, 14]. This dilemma is further highlighted by the fact that in several clinical trial subgroup and meta‐analyses, it has been observed that older BC populations seem to have comparable treatment benefits and toxicities compared to younger patients, but real‐world data have shown older women to exhibit worse rates of survival compared to younger [6, 16]. Again, multiple factors are likely involved, but it is reasonable to expect that age‐ or frailty‐related comorbidities, competing causes of death and vulnerability to treatment‐related complications and socioeconomic factors, together with the lack of aging related guidelines and comprehensive geriatric assessments may play a major role [6].

Considering widespread and age group agnostic clinical uptake of CDKis, understanding of external validity of demographic, safety and efficacy data reported in pivotal clinical trials in real‐life aged and frail subpopulation remains limited. A recent study from the large real‐world Flatiron register provided data on survival and dose intensity of palbociclib in a large BC cohort, but real‐world data in age‐related subgroups and concerning age‐related comorbidities in patients using CDKis are very limited [17].

We studied the real‐world patient characteristics including age and age‐ and frailty‐related co‐morbidities of the BC patients prescribed with CDKis palbociclib, abemaciclib and ribociclib in a comprehensive nation‐wide register study. We compared the survival between different age groups and aimed to indirectly characterise the real‐world BC population in relation to the data reported in pivotal clinical trials, to provide contextualisation and to inform on the external validity and concordance of clinical trial populations of advanced BC patients using CDKis.

## 2 Methods

### 2.1 Study Cohort

**FIGURE 1:**
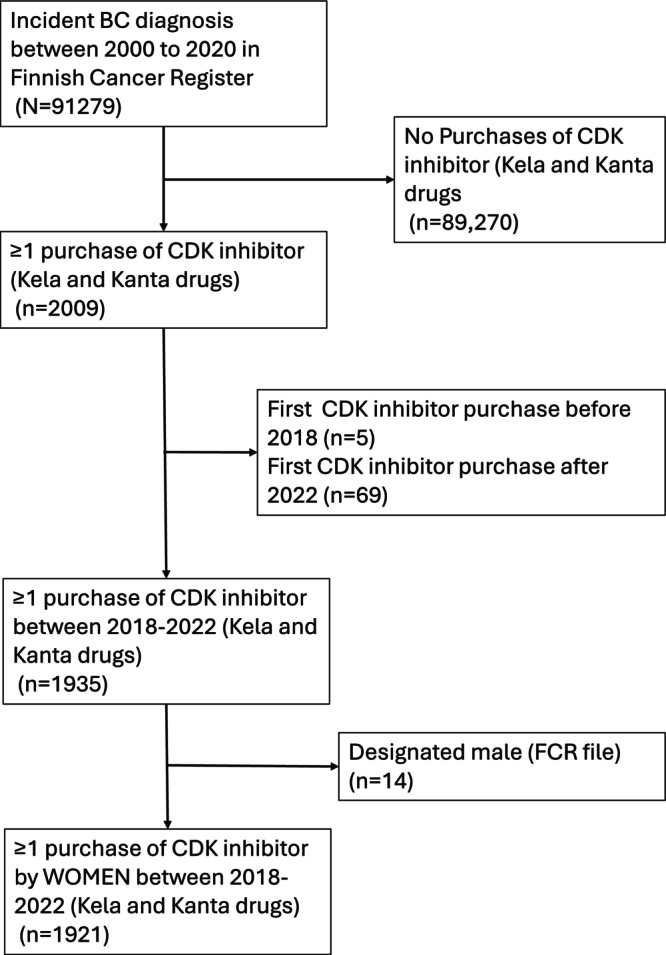
Identification of individuals and study cohort from the Finnish cancer registry and national prescription register.

Persons ≥ 18 years of age with their first diagnosis of BC were identified by the Finnish Cancer Registry (FCR) using the International Classification of Diseases (ICD) 10th version (C50) produced by the ICD O‐3 between 2000 and 2020 (*N* = 91 279) (Figure 1). The FCR is a national register of all cancer diagnoses in Finland (population 5.6 million) since 1953.

Personal identification numbers allow for linkage between multiple registers and the data were pseudonymised by the National Health and Social Data Permit Authority FinData before being released for research purposes. Ethics committee approval or informed consent was not required according to the Finnish legislation because pseudonymised, routinely collected register data were used, and the study participants were not contacted.

### 2.2 Drug Exposure

Data on reimbursed prescription drugs purchased in community pharmacies from 2018 to 2021 were extracted from the register of dispensed medicines reimbursable under the National Health Insurance (NHI) scheme, maintained by the Social Insurance Institution (SII). Information on all dispensed prescriptions, regardless of their reimbursement status, was also extracted from the KANTA Electronic Prescription Database from 2018 to 2022. CDKis were defined as Anatomical Therapeutic Chemical (ATC) substances as L01EF01, L01EF02, or L01EF03, and the corresponding older codes used before 2021 (Table S1). The recorded date of the first purchase of a CDKi is considered the index date.

Concomitant antihormonal drug prescriptions for breast cancer from the same time periods and data sources were extracted from the same Prescription Register (2018–2021) and the KANTA Electronic Prescription Database (2018–2023). The use of specific antihormonal drug substances, defined by 7‐character ATC codes (Table S1), for the time from first BC diagnosis to the index date and in the 1‐year follow‐up from the index date was assessed.

### 2.3 Comorbidities

Data on comorbidities was gathered from The Care Register for Health Care (CRHC) (maintained by The Finnish Institute for Health and Welfare (THL)), the Prescription Register, and the Special Reimbursement Register (maintained by SII). The CRHC contains diagnoses from both inpatient stays and outpatient visits to specialized healthcare. The Special Reimbursement Register contains data on entitlement to special reimbursement for chronic diseases. The prevalences of asthma/COPD, cardiovascular diseases, stroke, diabetes, osteoporosis, hip fractures, major cognitive disorders and severe mental and behavioral disorders were analysed. Analysis was conducted for all CDKi users, and separately for age groups ≥ 65 years and < 65 years of age, and for abemaciclib, palbociclib and ribociclib, respectively. The definition and data source for each comorbidity is described in detail in Table S1.

### 2.4 Survival

The date of death and underlying cause of death were obtained from the Causes of Death Register, maintained by Statistics Finland. Survival data ended on December 31, 2023. The median follow‐up time in the mortality analysis was 25 months (range: < 1 month–58 months).

### 2.5 Statistical Analyses

Characteristics of CDKi initiators and between the drug substances were compared using Chi‐squared for categorical variables. For continuous variables, summary statistics and Kruskal‐Wallis test were applied for non‐normally distributed variables (age and time from first breast cancer diagnosis). For estimation of survival analyses, the Kaplan–Meier survivor function was used and adjusted for comorbidities. Survival times are presented in months. To test differences between groups with respect to survival, the log‐rank test was used. *p*‐values ≤ 0.05 were considered statistically significant. All statistical analyses were performed with STATA 16.0 (STATACorp, College Station, TX, USA).

## 3 Results

### 3.1 Study Cohort Characteristics

Altogether 1921 women initiated treatment with any CDKi during the study period (Table 2). 91.5% (*n* = 1757) of individuals were prescribed palbociclib, while 111 abemaciclib and 53 ribociclib users were identified (Figure 1, Table 1). The median time since the first BC diagnosis, indicating the time of diagnosis of advanced/metastatic disease from the primary BC, was 7.5 years (Table 1). Per calendar year, the highest prescription numbers were in 2019, and for palbociclib (Table 2).

**TABLE 1:** Demographic characteristics of CDKi users^a^ and characterisation of study cohort per product.

	Any CDK inhibitor	Abemaciclib	Palbociclib	Ribociclib	P (difference between abema‐, palbo‐, ribococlib)
Number of individuals *N* (%)	1921	111 (5.8)	1757 (91.5)	53 (2.8)	
Cohort start date (minimum)	04/2018	07/2020	04/2018	01/2019	
Cohort end date (maximum)	12/2022	12/2022	12/2022	12/2022	
Age median [min (IQR) max]	66.9 [28 (57.8–73.8) 92]	66.6 (56.9–74.6)	66.9 (58–73.7)	66.1 (54.8–74)	0.87
Age category *n* (%)					0.88
< 65	829 (43.2)	48 (43.2)	757 (43.1)	24 (45.3)	
65–74	694 (36.1)	38 (34.2)	639 (36.4)	17 (32.1)	
75–84	365 (19.0)	> 20 (< 20.0)	< 340 (< 20.0)	12 (22.6)	
> = 85	33 (1.7)	< 5 (< 3)	> 25 (< 2.0)	0	
Time since first (local/advanced) breast cancer diagnosis (years, median, IQR)	7.5 (3.3–12.5)	7.7 (4.5–13.6)	7.5 (3.2–12.5)	7.1 (4.6–10.9)	0.14

^a^ All individuals with at least one purchase of abemaciclib/palbociclib/ribociclib in the database between 01/01/2018 and 31/12/2022, with breast cancer diagnosis. Age at initiation that is, at 1st purchase.

**TABLE 2:** Proportion of ademaciclib, palbociclib and ribociclib initiations out of all CDKi initiations per calendar year Number and proportion of patients^a^ by calendar and starting year.

Proportion of initiators by year *n* (%)	Abemaciclib	Palbociclib	Ribociclib
2018	0	125 (7.1)	< 5 (< 5)
2019	0	669 (38.1)	< 5 (< 5)
2020	5 (4.5)	428 (24.4)	< 5 (< 6)
2021	51 (46)	331 (18.8)	7 (13.2)
2022	55 (49.6)	204 (11.6)	41 (77.4)

^a^ 1st purchase that is, treatment initiations.

### 3.2 Age Categories in Patients Prescribed With CDKis

**FIGURE 2:**
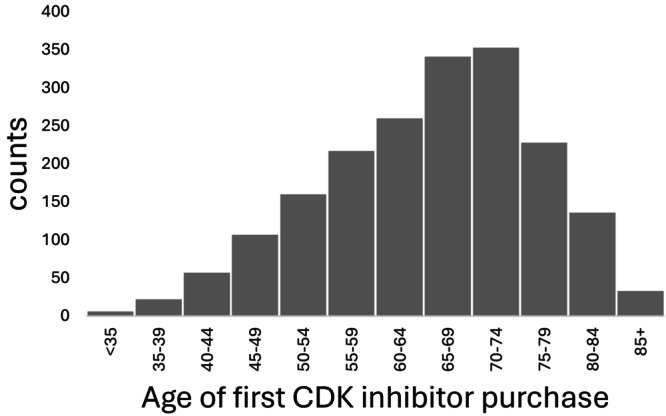
Age distribution of individuals prescribed with any CDKi during the study period.

The median age at CDKi treatment initiation was 66.9 years (Interquartile range (IQR) 57.8–73.8), and there was no significant difference between the CDKis prescribed. The median ages were 66.6 (IQR 56.9–74.6), 66.9 (IQR 58–73.7) and 66.1 (IQR 54.8–74.0) years for abemaciclib, palbociclib and ribociclib, respectively (Table 1). Age distribution of individuals is shown in Figure 2.

43.2% of CDKi users were < 65 years of age, 36.1% 65–74 years, 19.0% 75–84 years and 1.7% 85 years or older (Table 1). There were no differences in the age category distribution between individuals prescribed with palbociclib, abemaciclib or ribociclib (Table 1).

### 3.3 Comorbidities and Comedications

When all individuals with at least one purchase of abemaciclib/palbociclib/ribociclib in the database between 01/01/2018 and 31/12/2022 with a BC diagnosis were analysed, the highest prevalences were observed in cardiovascular diseases and diabetes, 22.3%, and 13.9%, respectively (Table 3). The prevalence of cardiovascular diseases and diabetes were significantly higher in individuals ≥ 65 years of age versus < 65 years of age using any CDKi (32.3% vs. 9.1%, *p* < 0.001, and 16.9% vs. 9.9%, *p* < 0.001, respectively). The prevalence of asthma/COPD, osteoporosis, hip fractures, major cognitive disorders, stroke and severe mental and behavioral disorders were low, with no significant differences between age groups (Table 3).

**TABLE 3:** Prevalence of concomitant diseases in CDKi users^a^ per product and age groups. Data are given as *n* (%).

Concomitant disease/co‐morbiity	Any CDK inhibitor *n* = 1921 *N* (%)	Abemaciclib	Palbociclib	Ribociclib	*p*‐value
Asthma/COPD	149 (7.8)	9 (8.1)	135 (7.7)	5 (9.4)	0.89
< 65 years	67 (8.1)				0.64
> = 65 years	82 (7.5)				
Cardiovascular diseases	428 (22.3)	26 (23.4)	398 (22.6)	4 (7.6)	0.032
< 65 years	75 (9.1)				< 0.001
> = 65 years	353 (32.3)				
Stroke	4 (0.2)	0	4 (0.2)	0	0.83
< 65 years	0				0.83
> = 65 years	4 (0.4)				
Diabetes	267 (13.9)	19 (17.1)	242 (13.8)	6 (11.3)	0.53
< 65 years	82 (9.9)				
> = 65 years	185 (16.9)				< 0.001
Osteoporosis	4 (0.2)	0	4 (0.2)	0	0.83
< 65 years	0				0.08
> = 65 years	4 (0.2)				
Hip fractures	6 (0.3)	< 5 (2.0)	5 (0.3)	< 5 (< 2.0)	0.49
< 65 years	< 3				0.63
> = 65 years	> 3				
Major Cognitive disorders	5 (0.3)	< 5 (< 2.0)	< 5 (< 0.5)	< 5 (< 2.0)	0.004
< 65 years	< 3				0.3
> = 65 years	> 3				
Severe mental and behavioral disorders	10 (0.5)	0	10 (0.6)	< 5 (< 2.0)	0.63
< 65 years	> 3				0.02
> = 65 years	< 3				

^a^ All individuals with at least one purchase of abemaciclib/palbociclib/ribociclib in the database between 01/01/2018 and 31/12/2022, with breast cancer diagnosis. Concomitant diseases at 1st purchase, that is, “user” defined at treatment initiation.

The most frequent antihormone agents prescribed in combination with CDKis in the entire cohort were fulvestrant (43.1%) and letrozol (42.8%), followed by exemestane, anastrozol, and tamoxifen (Table 4). The majority of individuals had a previous antihormone treatment for metastatic BC before CDKi initiation (Table 4).

**TABLE 4:** Antihormonal medications/combination partners of CDKi users per product and age group before and at/after^a^ first CDKi purchase.

Antihormonal medications *n* (%)	Any CDK inhibitor	Abemaciclib	Palbociclib	Ribociclib	*p*‐value
Anastrozol					
Before	383 (20)	24 (21.6)	345 (19.6)	14 (26.4)	0.43
At/after	71 (3.7)	5 (4.5)	63 (3.6)	6 (5.6)	0.67
Letrozol					
Before	1597 (83.1)	96 (86.5)	1459 (83)	42 (79.3)	0.48
At/after	822 (42.8)	40 (36.0)	763 (43.4)	19 (35.9)	0.18
Exemestane					
Before	423 (22)	36 (32.4)	374 (21.3)	13 (24.5)	0.02
At/after	330 (17.2)	24 (21.6)	299 (17.0)	7 (13.2)	0.34
Fulvestrant					
Before	478 (24.9)	30 (27)	434 (24.7)	14 (26.4)	0.83
At/after	828 (43.1)	43 (38.8)	760 (43.3)	25 (47.2)	0.54
Tamoxifen					
Before	1113 (57.9)	64 (57.7)	1019 (58)	30 (56.6)	0.98
At/after	55 (2.9)	< 3 (< 2.0)	52 (3.0)	< 3 (< 2.0)	0.71

^a^ Before refers to medications purchased between the first identified breast cancer diagnosis (earliest January 1, 2000) and before the first identified CDKi drug purchase. At/after refers to a one‐year follow‐up period of the index date that is, concomitant use.

### 3.4 Overall Survival in Different Age Categories of CDKi Users

**FIGURE 3:**
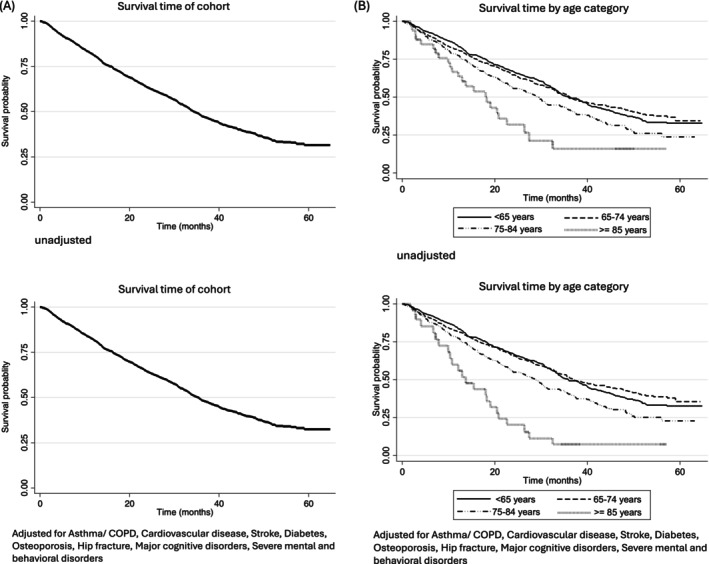
Kaplan–Meier curves of overall survival in all CDKi users (A) and different age categories of CDKi users (B).

To analyse the differences in the survival of patients using CDKis between age groups, we analysed OS in the study population and in the above‐defined age cohorts. During the study period, 1023 deaths occurred in the cohort and higher mortality was observed in older age groups. The median survival in the entire cohort was 25.3 months, 27.7 months in < 65 years of age, 25.1 months in individuals aged 65–74 years, 21.4 months in individuals aged 75–84 years and 15.4 months in individuals 85 years or older (Figure 3, Table 5). BC was reported as an underlying cause of death in 97% of the survival events (Table S2).

**TABLE 5:** Median survival^a^ of all CDKi users per age group.

Survival (2018–2023)	All age groups	< 65 years	65–74	75–84	> = 85	P (difference between palbo‐, adema‐, and ribociclib); difference with age groups
Events/number of cases						
All ciclibs	1023/1921	439/829	346/694	214/365	24/33	0.006
Abemaciclib	32/111	13/48	< 10/38	10/22	< 3/3	0.242
Palbociclib	977/1757	416/757	336/639	202/331	23/30	0.008
Ribococlib	14/53	10/24	< 3/17	< 3/12	No obs	0.069
Median survival (months)						
All ciclibs	25.3	27.7	25.1	21.4	15.4	
Abemaciclib	19.2	19	20.4	18.7	20.8	
Palbociclib	26.8	29.2	26.7	22.9	14.6	
Ribococlib	15.6	17.9	15.3	15.3	No obs	

^a^ Median survival from 1st purchase in all individuals with at least one purchase of abemaciclib/palbociclib/ribociclib in the database between 01/01/2018 and 31/12/2023, with breast cancer diagnosis.

## 4 Discussion

Clinical management of malignancies in older adults is characterised by a complexity of tumour biology, prognosis of the malignancy together with comorbidities, frailty, and patient preferences [18]. Treatment guidelines of cancer in older and frail patients emphasise tailored approaches [14, 19]. At the same time, cancer‐specific treatment guidelines, based on pivotal prospective clinical trials in the target indication, do not provide detailed specific guidance concerning patients with older age or frailty, and are applied across age groups in BC [11]. Together with the recognised underrepresentation of older women in pivotal clinical trials of BC, evidence‐based delineating of treatment decisions in older adults remains challenging [16].

To address the knowledge gaps concerning the real‐world use of the CDKis, current standard‐of‐care in advanced or metastatic BC, in older women, we analysed the demographics, age groups and co‐morbidities in a large retrospective nation‐wide cohort based on several registries. Our results provide indirect evidence on the external validity and contextualisation of pivotal clinical trials of CDKis in terms of age and co‐morbidities in patients with advanced or metastatic BC.

Our data indicate that in the real world, CDKis are used in older patient groups than represented in the pivotal clinical trials based on which they were authorised and introduced in clinical practice. The median age of patients in the initial pivotal trial of palbociclib that randomised 666 postmenopausal women with ER‐positive, HER2‐negative breast cancer, who had not had prior treatment for advanced disease, to receive palbociclib plus letrozole or placebo plus letrozole, was 61 years [7]. The initial pivotal phase III studies of ribociclib and abemaciclib reported median ages of 62 and 59 years, respectively [7, 8, 9]. Consequently, our data with a median age of 66.9 years indicates that the median age of those for whom CDKi treatment is initiated is 5–8 years higher than in these pivotal studies [3, 4, 5, 6, 7, 8, 9].

The median ages in our data set appear to reflect that of the overall hormone‐receptor positive metastatic BC population in the western countries, thereby supporting the generalisability of our findings [2, 3]. Previous analyses have suggested that a common cut‐off age for subgroup analyses in anti‐cancer clinical trials is 65 years, and that patients in age groups 75–84 of age are represented in such low proportions that clinically meaningful conclusions are difficult to draw [16]. Our data indicates that in a real‐world setting where the indication or reimbursement does not restrict the prescription in terms of age, over half of the patients using CDKis are over 65 years of age, and one fifth of the patients over 75 years of age. Interestingly, CDKis are also used in a small group of patients of over 85 years of age. This group of 1.7% of the entire CDKi user population, however, is also in our data too small to draw meaningful clinical conclusions, indicating that even in a reasonably large nation‐wide real‐world cohort, obtaining data that would be applicable in the clinical decision making in the very old age subgroups is inherently difficult. This would favour, in addition to higher cut‐offs, dedicated and tailored clinical studies in these groups.

Most observational studies have found that patients with cancer and comorbidities have a poorer survival than those without any comorbidity [20]. While survival rates vary by cancer type, cohort studies have reported 1.1‐ to 5.8‐fold higher mortality for BC patients with any comorbidity compared to patients with no comorbidity [21]. The very low accrual of older participants with major comorbidities or frailty in clinical trials remains a concern, and such patients are often excluded from trials based on the study protocol or investigator's judgement [1, 22]. Based on the inclusion/exclusion criteria, these patients have been excluded also from the pivotal trials of CDKis, and specific data on concomitant illnesses have not been reported. Therefore, we studied the prevalence of major age‐ and frailty‐related comorbidities in our cohort. Overall, our data indicate a low prevalence of these comorbidities, most notably very low of those of major cognitive disorders, hip fractures, osteoporosis, and stroke, at the time of treatment initiation with CDKis. This indicates good external validity of clinical trial populations in this respect and alleviates the concerns that most vulnerable older adults with major comorbidities and cancer would be overtreated while fit patients would be undertreated [23]. A statistically significant difference between individuals < 65 of age versus ≥ 65 years of age was noted in common diseases like cardiovascular diseases and diabetes. However, the prevalence of cardiovascular diseases, diabetes, and asthma was still slightly lower than in general female populations in both age groups under and over 65 years.

BC specific survival in older women has been reported to be similar to the survival in the general population irrespective of disease status, while an inferior OS has been reported in some studies [5, 6]. We observed a shorter OS in the oldest age groups of CDKi users, while the differences between the three younger age groups were smaller. Of note, the median OS from treatment initiation in our study cohort was 25.3 months, which is shorter than that reported from the final analyses of clinical trials or other large real‐world cohorts [17]. Although the follow‐up time is shortened by administrative censoring, the differences between age groups arising already within this time window may indicate the selected nature of the pivotal trial populations compared to a real‐world population. These findings underline the need for broader inclusion of individuals, particularly over 75 years of age in clinical studies to improve external validity, and for prospective studies to ensure that efficacy and safety of CDKis are maintained in older age groups.

Our findings need to be interpreted in the context of duration of follow‐up, together with the limitations in isolating purely BC specific deaths in registry data [24]. Notably though, BC was reported as the underlying cause of death in 97% of events in our data set, suggesting non‐BC causes to be a minority. Our survival findings may also be impacted by the time window of the cohort because patients who initiated CDKis soon after marketing authorisation are more likely to represent later treatment lines and heavily pretreated patients with a more advanced disease. This is supported by previous anti‐hormone treatments in the majority of CDKi initiators in this study, and fulvestrant being the most frequent product used in combination, as fulvestrant is indicated in patients with a previous antihormonal regimen for metastatic BC only.

In the Finnish drug reimbursement policy reimbursement of CDKis is not restricted in terms of age or comorbidities. Therefore, our data is expected to reflect the clinical decision‐making well. Equally, the representativeness of our study population is likely high, as together with high coverage of registries the registers capture both reimbursed and nonreimbursed dispensings. Finally, it is justified to assume that as our data is on actual purchase records of the products, the patients included in the data set represent actual users of CDKis. It is also noteworthy that our cohort is homogenous in terms of treatment aim and indication, that is, consisting of advanced or metastatic hormone‐receptor positive BC patients only, as the adjuvant and neoadjuvant indications and reimbursement of abemaciclib and ribociclib were approved after the study period ended.

In conclusion, the median age of real‐world patients using CDKis was higher than that reported in pivotal clinical trials, and the survival in the oldest age group was lower, suggesting challenges in extrapolating the findings of pivotal trials to real‐world settings. Nearly one fifth of our cohort were over 75 years old, favouring a broader inclusion of this age group in future BC pivotal trials to improve external validity. The low prevalence of co‐morbidities and very low prevalence of frailty‐related diseases in real‐world users alleviates the concerns of overtreatment and external validity of pivotal clinical trials in terms of frailty.

## Author Contributions

B.R.: data analysis, methodology, writing; O.T.: data analysis, methodology, writing; L.P.: methodology, writing; T.V.: data analysis; A.P.: data Analysis; M.T.: methodology, writing; S.H.: data analysis, methodology, writing; A.‐M.T.: data analysis, methodology, writing.

## Funding

This work was supported by Strategic Research Council Finland number 358417 (to M.T.). The remaining authors have nothing to report.

## Ethics Statement

Ethics committee approval or informed consent was not required according to the Finnish legislation because pseudonymised, routinely collected register data were used, and the study participants were not contacted.

## Conflicts of Interest

The authors declare no competing interests for this work. The views expressed in this article are the personal views of the authors and may not be understood or quoted as being made on behalf of or reflecting the position of the Finnish Medicines Agency or the European Medicines Agency, or one of their committees or working parties.

## Supporting information


**Table S1:** Identification of CDKi use, concomitant treatments, and comorbidities.

**Table S2:** Causes of death in the study cohort.

## Data Availability

The data that support the findings of this study are available from the corresponding author upon reasonable request.
