# Supplementary material for: Age, Age‐Related Comorbidities and Survival in Palbociclib, Ribociclib and Abemaciclib Users With Advanced Breast Cancer: A Nation‐Wide Retrospective Cohort Study
Source: Pharmacoepidemiol Drug Saf. 2026 Jun 16;35(7):e70416. doi: 10.1002/pds.70416 (PMC13270987; doi:10.1002/pds.70416)
Supplement: Supplementary file 1 — Table S1: Identification of CDKi use, concomitant treatments, and comorbidities. [file PDS-35-e70416-s002.docx]

**Supplementary Table 1. Identification of CDKi use, concomitant treatments, and comorbidities.**

| Variable | Data sources & coding | Time window |
| --- | --- | --- |
| CDKi user (any ciclib), separate variables for individual CDKis | **PR**  **KEPD**  **ATC: L01EF01(L01XE33)**  **L01EF02 (L01XE42)**  **L01EF03 (L01XE50)** | **Years 2018-2022** |
| Use of hormonal products, “combination partners of cdk inhibitors” | **PR**  **KEPD**  **ATC: L02BG04**  **L02BG03**  **L02BG06**  **L02BA01**  **L02BA03** | **First breast cancer diagnosis date until index date**  **(days- earliest January 1, 2000, -1)**  **One year from index date**  **(days 0-366)** |
| Major cognitive disorder | **CRHC**  **ICD-10: F00-03, G30, G31**  **PR**  **ATC: N06DA, N06DX**  **SRR**  **SRR code: 307** | **CRHC & PR:**  **Five years before index date**  **(days -1827, -1)**  **SRR:**  **from database inception (1964) to the day prior to the index date** |
| Cardiovascular diseases | **CRHC**  **ICD-10: I10-15, I20-I25, I44-50**  **SRR**  **SRR codes: chronic heart failure (201), hypertension (205), Chronic coronary artery disease and lipid metabolism disorder associated with chronic coronary artery disease (206), Chronic cardiac arrhythmias (207), dyslipidemia in chronic coronary artery disease (213), clopidogrel (280), anticoagulants (290), (351)** | **CRHC:**  **Five years before index date**  **(days -1827, -1)**  **SRR:**  **from database inception (1964) to the day prior to the index date** |
| Asthma/chronic obstructive pulmonary disease | **CRHC**  **ICD-10: J40-47**  **SRR**  **SRR code: 203** | **CRHC:**  **Five years before index date**  **(days -1827, -1)**  **SRR:**  **from database inception (1964) to the day prior to the index date** |
| Stroke (stroke) | **CRHC**  **ICD-10: I60-I64, I69** | **Five years before index date**  **(days -1827, -1)** |
| Diabetes | **CRHC**  **ICD-10: E10-E14**  **PR**  **ATC: A10 excluding A10BX01 (guargum)**  **SRR**  **SRR code: 103, 215, 285,** | **CRHC & PR:**  **Five years before index date**  **(days -1827, -1)**  **SRR:**  **from database inception (1964) to the day prior to the index date** |
| Osteoporosis | **CRHC**  **ICD-10: M80-81** | **Five years before index date**  **(days -1827, -1)** |
| Hip fractures | **CRHC**  **ICD-10: S72.0, S72.1, S72.2** | **Five years before index date**  **(days -1827, -1)** |
| Severe mental and behavioral disorders | **CRHC**  **ICD-10: F20-F31 F32.3 F33.3** | **Five years before index date**  **(days -1827, -1)** |
| Breast cancer as underlying cause of death | **Causes of death register**  **ICD-10: C50** | **index date to end of follow-up (December 31, 2023)** |

**Abbreviations:** ATC, Anatomical Therapeutic Chemical; CDKi, Cyclin-dependent kinase inhibitors; CRHC, Cater register for health care; ICD-10, International Statistical Classification of Diseases and Related Health Problems 10^th^ Revision; KEPD, Kanta Electronic Prescription Database PR, Register of dispensed medicines reimbursable under the National Health Insurance (NHI) scheme (previously known as the Prescription register); SRR, Special Reimbursement Register;
