# Supplementary material for: Age, Age‐Related Comorbidities and Survival in Palbociclib, Ribociclib and Abemaciclib Users With Advanced Breast Cancer: A Nation‐Wide Retrospective Cohort Study
Source: Pharmacoepidemiol Drug Saf. 2026 Jun 16;35(7):e70416. doi: 10.1002/pds.70416 (PMC13270987; doi:10.1002/pds.70416)
Supplement: Supplementary file 2 — Table S2: Causes of death in the study cohort. [file PDS-35-e70416-s001.docx]

|  | **Any CDKi** | **Abemaciclib** | **Palbociclib** | **Ribociclib** |
| --- | --- | --- | --- | --- |
| Breast cancer (C50) as underlying cause of death | 988 | 31 | 943 | 14 |
| Number of individuals that died | 1023 | 32 | 977 | 14 |
| Percentage (%) of breast cancer deaths of all deaths | 97 | 97 | 97 | 100 |

**Supplementary Table 2. Causes of death in the study cohort.**

**Abbreviations:** CDKi, Cyclin-dependent kinase inhibitor
